# Selective Catalytic Epoxidation–Hydration of α-Pinene with Hydrogen Peroxide to Sobrerol by Durable Ammonium Phosphotungstate Immobilized on Imidazolized Activated Carbon

**DOI:** 10.3390/nano13091554

**Published:** 2023-05-05

**Authors:** Min Zheng, Xiangzhou Li, Youyi Xun, Jianhua Wang, Dulin Yin

**Affiliations:** 1 College of Material Science and Engineering, Central South University of Forestry and Technology, Changsha 410205, China; 2 College of Physics and Chemistry, Hunan First Normal University, Changsha 410205, China; 3 National & Local Joint Engineering Laboratory for New Petro-Chemical Materials and Fine Utilization of Resources, College of Chemistry and Chemical Engineering, Hunan Normal University, Changsha 410081, Chinadulinyin@126.com (D.Y.)

**Keywords:** sobrerol, α-pinene, catalytic synthesis, ammonium phosphotungstate, activated carbon

## Abstract

It is presented that the activated carbon was carboxylated with hydrogen peroxide and then acylated with 2-methylimidazole to prepare the porous carbon support with a surface imidazolated modification. Through the adsorption of phosphotungstic acid on the fundamental site of an imidazolyl group and then adjusting the acid strength with the ammonia molecule, a catalytic carbon material immobilized with ammonium phosphotungstate (AC-COIMO-NH_4_PW) was obtained, which was used to catalyze a one-pot reaction of convenient α-pinene and hydrogen peroxide to sobrerol. The bifunctional active site originated from the dual property of ammonium phosphotungstate, as the oxidant and acid presenting a cooperatively catalytic performance, which effectively catalyzes the tandem epoxidation–isomerization–hydration of α-pinene to sobrerol, in which the solvent effect of catalysis simultaneously exists. The sobrerol selectivity was significantly improved after the acid strength weakening by ammonia. Monomolecular chemical bonding and anchoring of ammonium phosphotungstate at the basic site prevented the loss of the active catalytic species, and the recovered catalyst showed excellent catalytic stability in reuse. Using acetonitrile as the solvent at 40 °C for 4 h, the conversion of α-pinene could reach 90.6%, and the selectivity of sobrerol was 40.5%. The results of five cycles show that the catalyst presents excellent stability due to the tight immobilization of ammonium phosphotungstate bonding on the imidazolized activated carbon, based on which a catalytic-cycle mechanism is proposed for the tandem reaction.

## 1. Introduction

Sobrerol, a monocyclic monoterpenoid dihydric alcohol, was found to have specific preventive and therapeutic effects on cardiovascular diseases, which also could be used to treat secret otitis media in childhood [1]. With the advancement of medical standards and detection means, sobrerol was found to be a novel and promising therapeutic agent for human colorectal cancer; it can restore the activity and efficacy of cetuximab in ras-mutated tumors [2]. It is particularly desirable to develop higher-value drugs for combination treatment with sobrerol. With the spread of COVID-19, many cases showed that herbal medicines can interfere with COVID-19 pathogenesis by inhibiting SARS-CoV-2 replication and entry to host cells [3]. Sobrerol and monoterpenoids have attracted attention to treat pulmonary hypertension [4]. It is particularly desirable to develop multi-functional and efficient drugs for combination treatment with sobrerol. In addition, it has a particular application value in perfume and fine chemical industries. It can also be used as sobrerol acrylate [5] or sobrerol lactone [6] monomers for sustainable renewable polymers with antibacterial functions.

Although sobrerol can be extracted from some plants, the content is very low. For instance, the content of sobrerol in Teucrium scordium was detected at 2.26% by automatic thermal desorption-GC-MS [7], 0.63% in Mentha spicata essential oil by GC-MS [8], even at a very low yield of 7 mg/kg from the separation of sobrerol in the fruits of Illicium lanceolatum [9]. There is also a strategy for synthesizing sobrerol starting from methyl 3,5-dihydroxy-4-methyl benzoate in eight steps with an overall yield of 26% [10]. Therefore, the greener semisynthesis of sobrerol has been considered in the academia and industry from cheap α-pinene, the main component of the rich natural-resource turpentine, with a productivity of around 330,000 tons per year worldwide [11]. Based on the diversity of its molecular structure and reactive position, the selectively catalytic conversion of α-pinene as a synthetic block for fine monoterpenoid chemicals has been continuously promoted around the world, such as hydration [12,13,14,15]. The catalytic epoxidation of α-pinene, which provides α-epoxypinane as a synthetic intermediate for sobrerol, is still being explored to substitute for stoichiometric organic peroxide oxidation method in a different group, such as microcrystalline cellulosic salen complexes [16], Novozym435 [17], dioxo-molybdenum (VI)-based MOFs [18] or sieves Mn/SAPO-34 [19]. The stories on the achievement of the green synthesis process of sobrerol are mainly to explore new catalytic systems for the isomerization of α-pinene oxide to sobrerol, such as mesoporous molecular sieves Sn/SiO_2_ [20], vanadium-containing nickel phosphate molecular sieves [21], even soluble phosphotungstic acid [22] or solid Cs_2.5_H_0.5_PW_12_O_40_ acid salt [23].

Obviously, the one-step synthesis of sobrerol from α-pinene with reusable solid catalyst has the advantages of a green process. It was earlier found that sobrerol can be preciously obtained through biologically converting α-pinene by microorganism Armillariella mellea [24], which was incubated in 0.2% α-pinene for 4–5 days with a yield of sobrerol of 20%. Recently, interesting biomimetic catalytic dihydroxylation of α-pinene with H_2_O_2_ to sobrerol in one pot was delivered by a water-soluble peroxo vanadium peracid under benign reaction conditions [25]. There are still no data given on the reuse of catalyst, the separation of the reaction mixture and the purification of the product sobrerol. Developing of a highly efficient heterogeneous process using an easily recyclable solid catalyst would promote the improvement to the green preparation of sobrerol from α-pinene and the excellent utilization of turpentine resources.

In our recent work, it was published that hydroxyl-assisted selective epoxidation of perillyl alcohol with hydrogen peroxide is catalyzed by vanadium-substituted phosphotungstic acid hinged on imidazolyl activated carbon. [26]. In this work, by way of acylation with imidazole on carboxylated activated carbon, the basic activated carbon carrier is constructed, with the imidazole group as the monomolecular base site for the chemical adsorption of phosphotungstic acid. After ammonia fumigation, the phosphotungstic acid immobilized on the imidazolized activated carbon is transformed into strongly anchored ammonium phosphotungstate, which could catalyze the selective tandem epoxidation–isomerization–hydration of α-pinene to sobrerol under mild reaction conditions. The investigation of catalytic stability using five cycles of catalyst reuse tests, an efficient measure for recovering catalytic performance of a fresh catalyst is provided, and a catalytic cycle mechanism is proposed for the tandem reaction.

## 2. Experimental Procedure

### 2.1. Materials and Reagents

Phosphotungstic acid (HPW) was purchased from Aladdin, 99% α-pinene was provided by Yongzhou Lihao Technology Co., Ltd. (Yongzhou, China), and 30% hydrogen peroxide solution was provided by Baling Branch, SINOPEC. Other reagents were purchased from Sinopharm Chemical Reagent Co., Ltd. (Shanghai, China). All chemicals were not further purified before using.

### 2.2. Catalyst Preparation

**Scheme 1:**
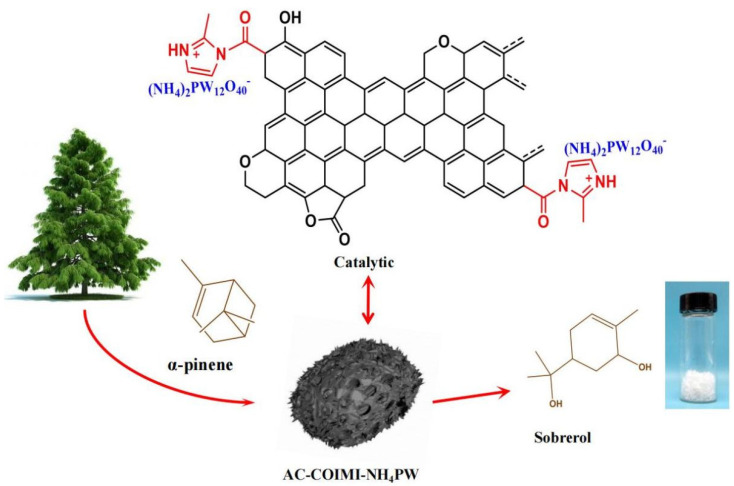
The preparation procedure and application of AC-COIMI-NH_4_PW.

According to the previous operation [27], the preparation of an imidazolized nitrogen-doped activated-carbon-supported phosphotungstic-acid catalyst AC-COIMI-NH_4_PW is shown in Scheme 1 and the operational details was carried out: the activated carbon (AC) was oxidized with hydrogen peroxide, replacing corrosive and polluting nitric acid oxidation [28]. Activated carbon (10 g), 30% H_2_O_2_ (100 mL,) and 12 mol·L^−1^ H_2_SO_4_ (10 mL) were added to a three-necked flask equipped with a condenser and thermometer and stirred in an oil bath at 70 °C for 2 h. Then the reaction mixture was filtered, washed repeatedly with deionized water until there was no SO_4_^2−^, and the wet solid material was dried in an oven at 120 °C for 4 h. The collected carboxylated activated carbon is marked AC-O. The acyl imidazolized activated carbon AC-COIMI was obtained by co-heating AC-O with 2-methylimidazole in a solvent-free manner, followed by adsorption of HPW to obtain the catalyst AC-COIMI-HPW. In a sealed dryer, the acidic site of AC-COIMI-HPW was weakened by fumigation of ammonia volatilized from concentrated ammonia. In this way, the collected catalyst, ammonium phosphotungstate immobilized on AC-COIMI is designated as AC-COIMI-NH_4_PW, with a saturated adsorption phosphotungstic acid content of 0.10 mmol·g^−1^ in a typical catalyst.

### 2.3. Catalytic Materials Characterization

XRD patterns of the sample were recorded by a Bruker diffractometer with Cu Kα radiation and diffraction angle (2θ) ranging from 10° to 80°. FT-IR of the samples was collected by the KBr pellet technique on a Nicolet 370 infrared spectrophotometer in the range of 400–4000 cm^−1^. XPS of the materials was collected by a PHI VersaProbe 4 spectrometer (Kanagawa-ken, Japan)in the range of 0–1200 eV. The UV–Vis spectrum in the preparation of catalysts was measured by Shimadzu UV-2450 (Kyoto, Japan) in 190–900 nm. Inductively coupled plasma-emission (ICP) chemical analysis of W in the reaction liquids was carried out by ICAP 7200 spectrometer (Suzhou, China). SEM data of the sample were obtained by Zeiss Sigma 300 (Oberkochen, Germany) scanning electron microscope, with magnification ranging from 50 to 10,000 times.

### 2.4. Catalytic Performance Test

The catalytic evaluation was carried out in a two-necked flask by batch reaction operation. In a typical operation, α-pinene 10 mmol, solvent 10 mL and weighted catalyst were added into the flask, then heated to the specified temperature. When the temperature was stable, a quantitative H_2_O_2_ was added with 1 drop interval of 5 s by drip funnel. As required reaction time, 0.5 mL was sampled with a 1 mL syringe under constant stirring for the analysis of product composition. The qualitative analysis was run by Shimadzu GC-MS-QP2010, the quantitative analysis by Shimadzu GC- 2010, HP-5 capillary (0.32 mm × 30 m), temperature programmed operation 80 °C (2 min) → 10 °C·min^−1^ → 180 °C (5 min).

## 3. Results and Discussion

### 3.1. Characterization

**Figure 1:**
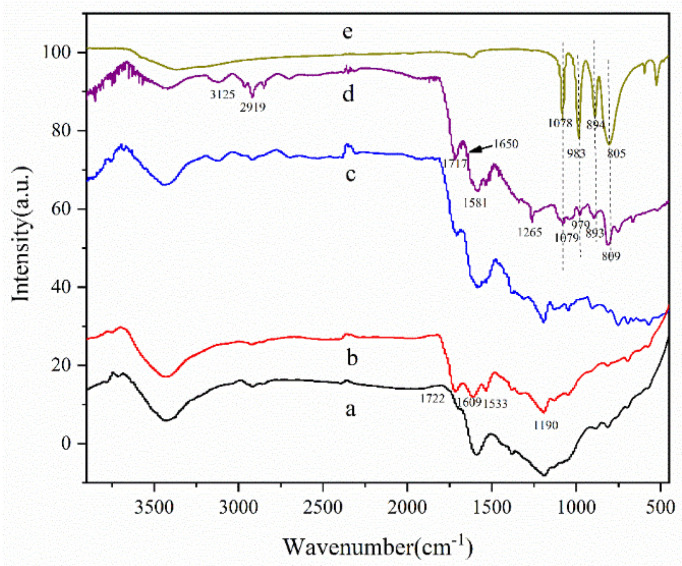
FT-IR of (**a**) AC-O, (**b**) AC-COIMI, (**c**) AC-COIMI-HPW, (**d**) AC-COIMI-NH_4_PW and (**e**) HPW.

Figure 1 shows the carbonyl stretching vibration peak at 1722 cm^−1^ from the imidazolized activated carbon AC-COIM. In addition, the absorption peaks at 1609 cm^−1^ and 1533 cm^−1^ which belong to the C=C stretching vibration zone of the fused-ring aromatic hydrocarbon skeleton in the activated carbon still maintain in the oxidized activated carbon. The peak at 1190 cm^−1^ is attributed to the C–O stretching vibration of the lactone group or phenolic hydroxyl group on the activated carbon. The absorption peaks in the above infrared images fully prove the existence of abundant functional groups on activated carbon, which also provides basic conditions for us to modify activated carbon by chemical methods. As compared with AC-COIMI, AC-COIMI- HPW retains its 2-methylimidazole structure and therefore presents the characteristic absorption of the imidazole skeleton. The peak at 3125 cm^−1^ belongs to the C-H stretching vibration on the unsaturated carbon of fused-ring aromatic hydrocarbons on the imidazole and methyl group. The absorption at 2919 cm^−1^ belongs to the N-H stretching vibration of the ammonium and imidazolium cation. The absorption at 1717 cm^−1^ still belongs to the stretching vibration of the carbonyl group, 1650 cm^−1^ represents the –C=N stretching vibration on the imidazole skeleton, and 1265 cm^−1^ is attributed to the C–N bending vibration peaks on the imidazole ring. The four main characteristic bands of pure phosphotungstic acid are located at 1078 (P–O_a_), 983 (W=O_d_), 894 (W–O_b_–W with co-angular octahedron) and 805 cm^−1^ (W–O_c_–W with co-lateral octahedral); the corresponding peaks can be found in AC-COIMI-HPW and AC- COIMI-NH_4_PW. The characteristic peak of heteropoly acid has a slight deviation, which is mainly because the lone pair of electrons in the organic cation extends into the inorganic framework of the heteropoly anion, which makes the formation of a strong electronic force, so that the characteristics of both sides and the absorptions shifted to a certain extent, and these shift changes indicate the interaction between the phosphotungstate anion and imidazolium cation [29]. A nonobvious Keggin structure absorption peak is observed in the catalyst, which confirms that the phosphotungstic acid species is highly dispersed.

**Figure 2:**
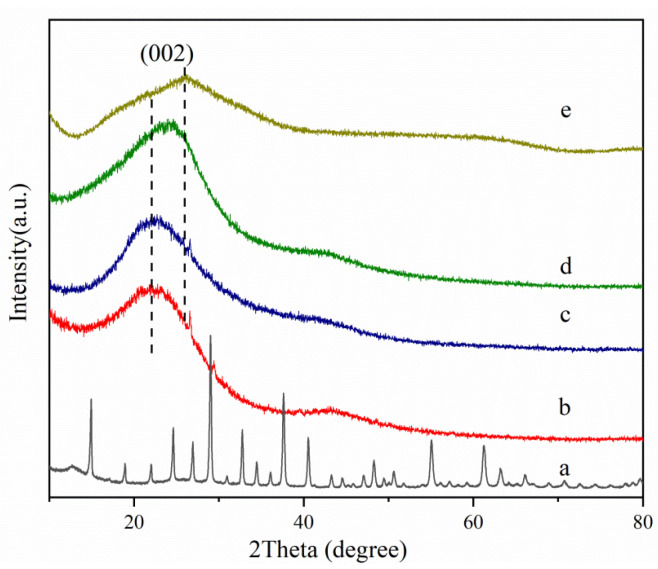
XRD of (**a**) HPW, (**b**) AC-O, (**c**) AC-COIMI, (**d**) AC-COIMI-HPW and (**e**) AC-COIMI-NH_4_PW.

The XRD pattern of HPW, AC, AC-O, AC-COIMI and AC-COIMI-HPW is shown in Figure 2, the Keggin cubic structure diffraction peaks of HPW are consistent with the reported results. It is shown that the characteristic diffraction peaks of HPW crystalline aggregates do not appear at all in AC-COIMI-HPW.

**Figure 3:**
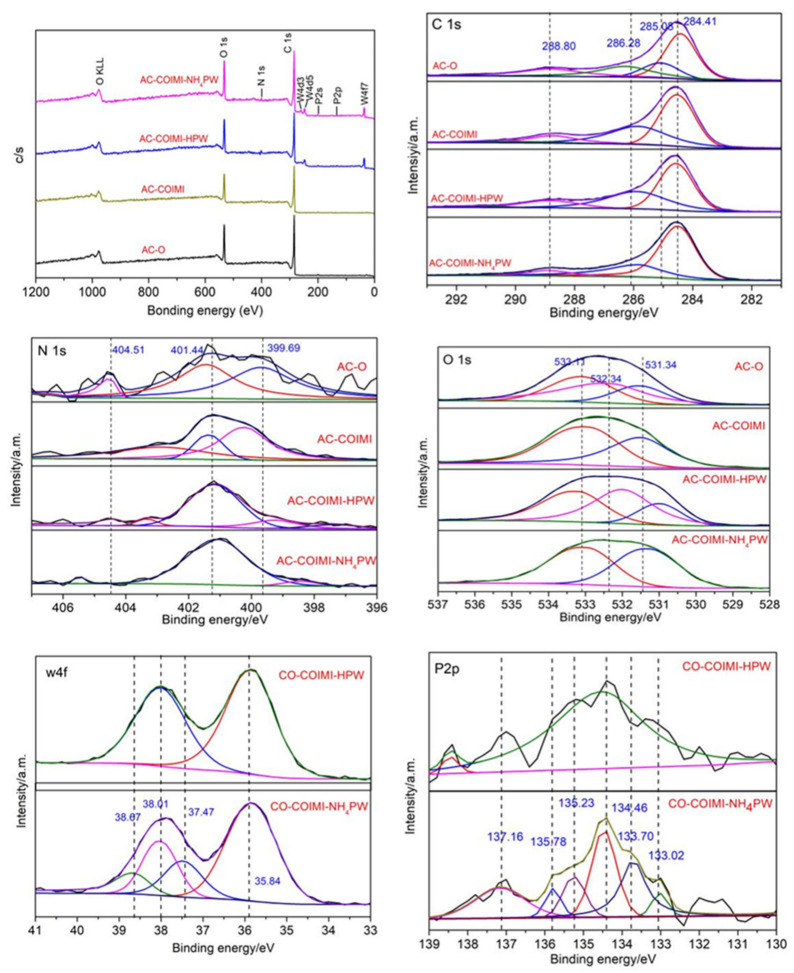
XPS of catalytic materials.

XPS data of the four catalytic materials, namely, AC-O, AC-COIMI, AC-COIMI-HPW and AC-COIMI-NH_4_PW, are presented in Figure 3. It is shown that N1s characteristic absorption appears in AC-COIMI from the imidazolization of AC-O. After HPW was immobilized on AC-COIMI by a single molecule, AC-COIMI-HPW showed obvious XPS absorption of W element, especially the absorption of W4f7. Due to the formation of NH_4_^+^ by H^+^ and NH_3_ on AC-COIMI-HPW, the XPS spectrum of W in the weak acidic AC-COIMI-NH_4_PW catalyst is stronger, and its W4d3 peak shape is more symmetrical than that of AC-COIMI-HPW, which indicates a reduction in the acid strength of the catalytic active center after ammoniation.

**Figure 4:**
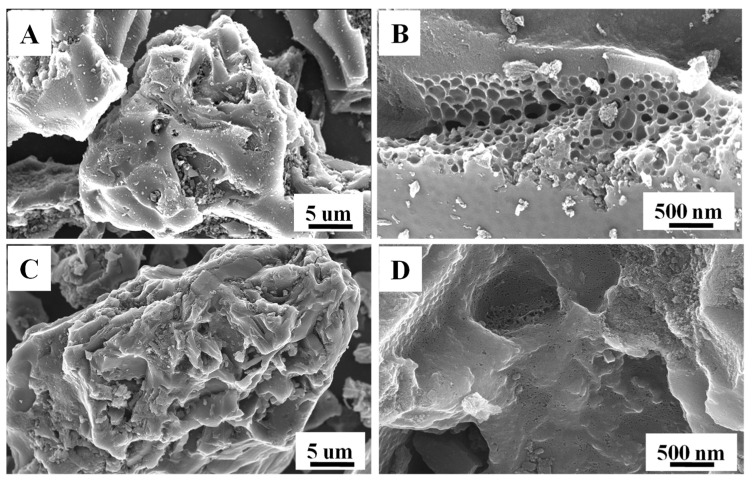
SEM of AC (**A**,**B**) and AC-COIMI-NH4PW (**C**,**D**).

SEM images in Figure 4 show that both AC (Figure 4A,B) and AC-COIMI-NH_4_PW (Figure 4C,D) exhibit typical amorphous carbon morphology. After oxidation treatment and imidazole functionalization, the adhered nanofragments or particles on the surface of AC have mostly disappeared(Figure 4C). The surface structure of AC-COIMI-NH_4_ PW appears more compact under SEM visualization (Figure 4D), which contributes to maintaining the particle stability of the catalyst in the operating environment.

### 3.2. Construction of Catalytic Sites for Tandem Epoxidation–Hydration

The catalytic reaction results of α-pinene with hydrogen peroxide in acetonitrile as solvent on different carbon-based materials are listed in Table 1. It can be seen that the oxidized products distribution of α-pinene in the presence of activated carbon (AC) is the same as the blank reaction by hydrogen peroxide, indicating that the oxidative catalysis of AC is almost negligible in the reaction system. When pre-oxidized activated carbon (AC-O) was used as a catalyst, which performed as an acid catalytic feature, the conversion of α-pinene was increased by nearly 10 percentage points relative to AC. Some monoterpene isomers and a small amount of monoterpene alcohols might be mainly formed through the isomerization of α-pinene catalyzed by the proton in the carboxyl group on AC-O. Once basic carbon material AC-COIMI from the imidazolization of carboxyl site on AC-O was added to the reaction flask as a catalyst, the conversion of α-pinene is lower than that of blank reaction; acid-catalyzed isomerization and hydration of α-pinene were almost completely inhibited.

Based on phosphotungstic acid (HPW), species not only can be provided with oxidative catalysis for the epoxidation of olefins, but also can be used for the acid catalytic rearrangement of α-pinene oxide to sobrerol [23], which was actually produced by the catalytic hydration of α-pinene oxide with water in the solvent. HPW was selected as the catalytic component used to construct carbon-based solid catalysts for tandem epoxidation–hydration of α-pinene to sobrerol.

The conversion of α-pinene was significantly increased to 89.3% with AC-COIMI-HPW as a catalyst, which shows that acid and oxidation catalysis coexist on AC-COIMI-HPW. However, the total selectivity of the direct isomerization and hydration of α-pinene exceeds half catalyzed by the acid site, sobrerol accounts for about one-quarter of products from the oxidation, and its selectivity is only 10.5%, which shows that the acid catalysis of AC-COIMI-HPW far exceeds its oxidative catalysis. The H^+^ acid strength of AC-COIMI-HPW should be modified to improve the selectivity of sobrerol.

**Figure 5:**
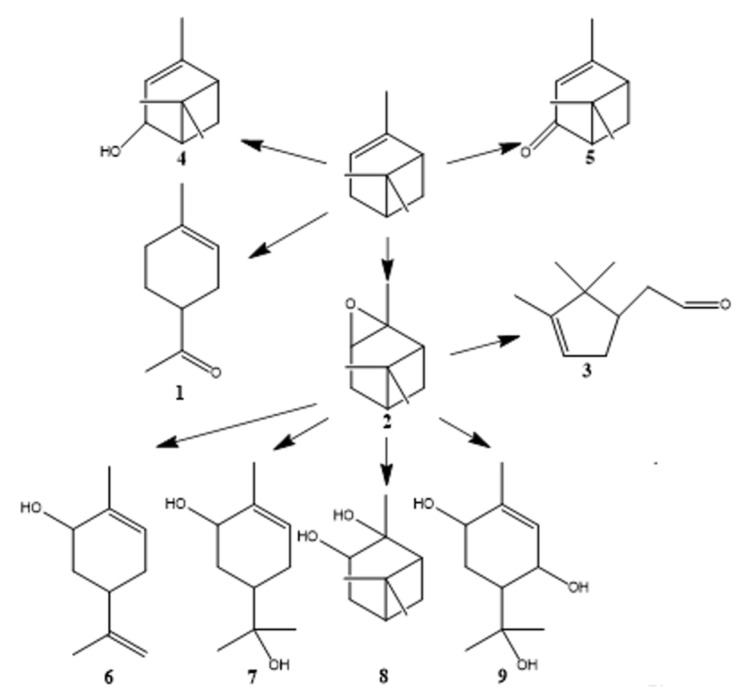
The formation path of oxidative products in α-pinene with H_2_O_2_.

Based on acid–base conjugation, the strong proton H^+^ acid site was converted into a weaker ammonium NH_4_^+^ acid site by the adsorption of ammonia gas NH_3_ on AC-COIMI-HPW to prepare the AC-COIMI-NH_4_PW catalyst. Under the same reaction conditions, the catalytic activity for the conversion of α-pinene changed little on AC-COIMI-NH_4_PW, but the total selectivity of α-pinene oxidative reaction path is increased significantly, and nearly half of them are epoxidation–hydration series products, in which the selectivity of sobrerol is raised four times to 40.5%. Based on the molecular structure characteristics of α-pinene combined with the role of a composite catalytic site on AC-COIMI-NH_4_PW, the proposed parallel and sequential reaction pathway of α-pinene epoxidation–hydration and oxidation by H_2_O_2_ is illustrated in Figure 5.

With AC-COIMI-NH_4_PW, the primary reaction of α-pinene is mainly the epoxidation of its double bond, then the intermediate α-pinene oxide **2** reacts with H_2_O to form 7, and a small part of α-pinene oxide directly converts to 3 and 6 through the different splitting and migration of the carbon frame in the rearrangement process catalyzed by the acid site, but 8 is formed by the direct catalytic addition of 2 with H_2_O. In parallel, the allylic oxidation of α-pinene with H_2_O_2_ at the C3 position produces 4 and 5. Product 9 is derived from the consecutive allylic oxidation of 7. It can be inferred that product 1, 4-acetyl-1-methyl-1-cyclohexene, with nine carbon atoms, should come from the oxidative degradation of the outer double bond of limonene formed by the isomerization of α-pinene.

### 3.3. Solvent Modification of Catalytic Performance

The results of solvents on the reaction of α-pinene with the hydrogen peroxide aqueous solution on AC-COIMI-NH_4_PW are listed in Table 2. The reaction is difficult to occur in the hydrophilic solvent absence, and α-pinene is slowly converted in different solvents. The order of α-pinene conversion is acetonitrile(ACN) > acetone > tetrahydrofuran(THF) > butanone > dioxane> acetic acid(HAC) > ethyl acetate(EA) > dichloromethane(DCM) ≈ solvent-free. From the dipole moment data in parentheses of some solvents in the table, it can be seen that there is no consistent trend correlation between catalytic performance of AC-COIMI-NH_4_PW and dipole moment. It is indicated that the solvent effect on the reaction system of α-pinene with aqueous hydrogen peroxide is significant, which may be comprehensively determined by the polarity, hydrophilicity and Lewis basicity of the solvent molecule.

The polarity of the solvent corresponds to the catalytic performance of COIMI-NH_4_PW active sites in the conversion of α-pinene. It is difficult to catalyze the conversion of α-pinene in a solvent with small polarity such as dichloromethane or ethyl acetate, whose solubility in water is also poor so that it is also difficult to improve the reaction substrate H_2_O_2_ transport to the catalytic active site in AC-COIMI-NH_4_PW. Unlike the isomerization of α-pinene, which might takes place in the presence of water or wet raw materials, over heterogeneous sol–gel Sn/SiO_2_ or Ce/SiO_2_ catalysts to give mainly sobrerol, using a weakly basic acetone as solvent [20], acetonitrile as a stronger basic solvent, in which the lone pair electrons on the nitrogen atom determine its Lewis basicity, is more conducive to selectively promote the tandem epoxidation–hydration of α-pinene and hydrogen peroxide aqueous solution on the surface of AC-COIMI-NH_4_ PW to sobrerol, which essentially seems to be due to the dihydroxylation of α-pinene with H_2_O_2_ in acetone by the soluble peroxo-vanadium acid as catalyst [25]. Notably, the allylic oxidation of α-pinene by oxygen-functionalizing of C2 is promoted on [NH_4_PW] sites in several oxygenated solvents with ester or ether bonds. The sum selectivity of **4** and **5** is about 45%, which means that the solvent not only promotes the three-phase mass transfer of “α-pinene–hydrogen peroxide–solid catalyst” in the reaction process, but also participates in the construction of catalytic sites in the reaction environment through hydrogen bonds or coordination bonds, thus regulating the selectivity of the oxidation pathway on the [NH_4_PW] sites implanted on the surface of the modified activated carbon.

**Figure 6:**
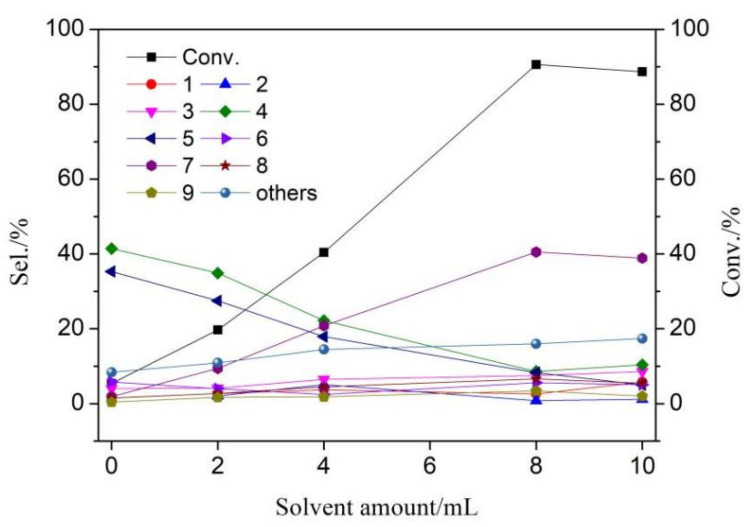
Effect of solvent amount on the reaction of α-pinene with H_2_O_2_. Reaction condition: α-pinene 10 mmol, catalyst 0.1 g, H_2_O_2_ 20 mmol, 40 °C, 3 h.

Figure 6 shows the effect of the amount of solvent acetonitrile, which contains a nitrile group and can provide a weak base and proton transfer, on the modification of catalytic performance in the tandem reaction of α-pinene on AC-COIMI-NH_4_PW. It could be seen that the amount of acetonitrile on the catalytic effect in the reaction of α-pinene with hydrogen peroxide is significant. Without solvent or with a small amount of acetonitrile, α-pinene is rarely converted, and a small amount of main products come from allyl oxidation of α-pinene. The ineffective decomposition of hydrogen peroxide is relatively violent, from which it can be observed that oxygen bubbles emerge from the reaction liquid. With the increase of the amount of acetonitrile, α-pinene can be better dissolved in the reaction solution system, and the decomposition of hydrogen peroxide also slows down, while the microcosmic environment of the reaction is modified to be more suitable for the tandem reaction, the conversion of α-pinene is significantly improved, and the epoxidation with subsequent ring-opening hydration of epoxy-bond become the main reaction paths. It is worth noting that an increase in the amount of acetonitrile does not translate to a better catalytic performance. Increasing the amount of acetonitrile in excess will reduce the concentration of the substrate in the reaction solution, and the conversion of pinene will be delayed. It can be deduced that the solvent, as an assistant, possesses the potential effect of regulating the catalytic performance from a quantitative to a qualitative change in the process of some fine catalytic synthesis.

### 3.4. Effect of Reaction Conditions on the Process

**Figure 7:**
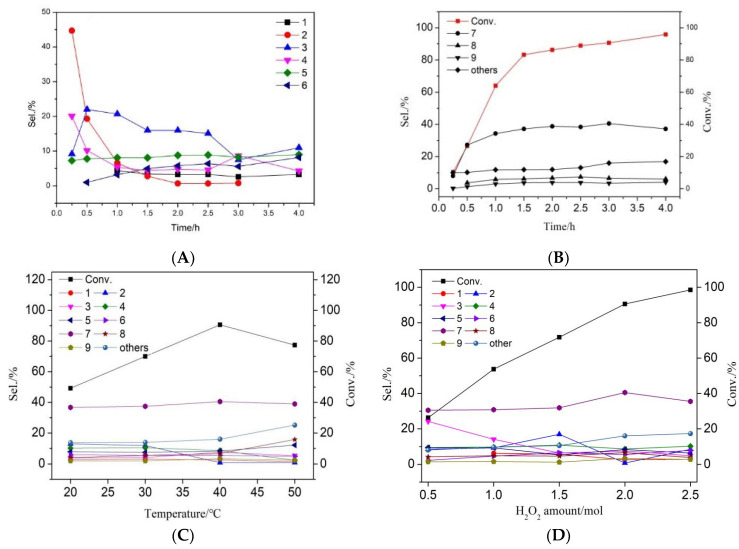
Effect of reaction conditions on the reaction of α-pinene with H_2_O_2._ Reaction condition: α-pinene 10 mmol, acetonitrile 8 mL, H_2_O_2_ 20 mmol, catalyst 0.1 g, 40 °C, 3 h.

In the reaction process of α-pinene and hydrogen peroxide within 4 h, the conversion of α-pinene and the selectivity of oxidative products can be seen in Figure 7A,B. It can be seen that AC-COIMI-NH_4_PW can efficiently catalyze this process and α-pinene can be continuously converted with the extension of reaction time, and almost completely oxidized with the highest conversion of 95.8%. From the epoxidation of α-pinene, the selectivity of 2,3-epoxypinane (**2**) continues to decline, showing the conversion characteristics of **2** as an intermediate product in a series of reactions. After 1.5 h, **2** selectivity is less than 1%, which indicates that once **2** is formed, it will be rapidly reacted into subsequent products. The selectivity of sobrerol (**7**), the target product from the hydration of **2** catalyzed by the acid site on AC-COIMI-NH_4_PW, reached the highest of 40.5% at 3 h, while the selectivity of other oxidation products remained unchanged through the hydration of **2**. However, **3** obtained from the acid catalytic rearrangement of **2**, which contains a more active aldehyde group, is further oxidized or converted into others. In the allylic oxidation of α-pinene, the data in Figure 6 show that the hydroxyl group of alcohol **4** can also be oxidized to ketone **5** over the catalyst AC-COIMI-NH_4_PW. It is further shown that the rationality of the catalytic main reaction pathway described in Figure 5 is due to the effect of time on the reaction of α-pinene with hydrogen peroxide over the catalyst.

Raising the reaction temperature will generally increase the reaction rate, but in the reaction of α-pinene and hydrogen peroxide on AC-COIMI-NH_4_PW, the conversion of α-pinene is the highest at 40 °C with the increase of temperature, while the conversion is reduced at 50 °C as can be seen in Figure 7C. It might be that with the acceleration of temperature, the invalid decomposition rate of hydrogen peroxide is more than its participation in the needed epoxidation or oxidation of α-pinene, reducing the effective concentration of hydrogen peroxide in the reaction system, so that the conversion of α-pinene is reduced, which means that increasing the reaction temperature would reduce the utilization efficiency of hydrogen peroxide in the process. With the increase of temperature, the selectivity of high-value **7** from the tandem epoxidation–hydration of α-pinene is slightly decreased, as is the selectivity of others, including **9** generated by the consecutive oxidation of **7**, **3**, **6** and **8** consecutively from α-pinene epoxidation of **2**, **4** and **5** from the allylic oxidation of α-pinene, which means that the activation energy of the reaction rate control step to produce other oxidizing by-products is higher in the one-step catalytic synthesis of α-pinene and hydrogen peroxide to **7**, and the lower reaction temperature is beneficial to improve the selectivity of **7.**

Figure 7D shows the effect of the ratio of hydrogen peroxide and α-pinene on the reaction results of the catalyst AC-COIMI-NH_4_PW. When the ratio is 0.5, 4-acetyl-1-methyl-cyclohexene and carvol were not detected, the main products are sobrerol and campholenic aldehyde. It is shown that the conversion of α-pinene is increased synchronously with increasing the ratio of hydrogen peroxide. The selectivity of campholenic aldehyde and other mono-oxygen products is decreased, the selectivity of sobrerol and poly-oxygen products is increased. When the ratio is 2, the conversion of α-pinene can reach 90.6%, and the selectivity of sobrerol can rise to 40.5%. Although the amount of hydrogen peroxide continues to increase, α-pinene can be almost completely converted, but the selectivity of sobrerol would gradually decrease, so the ratio of hydrogen peroxide to pinene is appropriate to be 2.

### 3.5. Catalytic Stability

**Figure 8:**
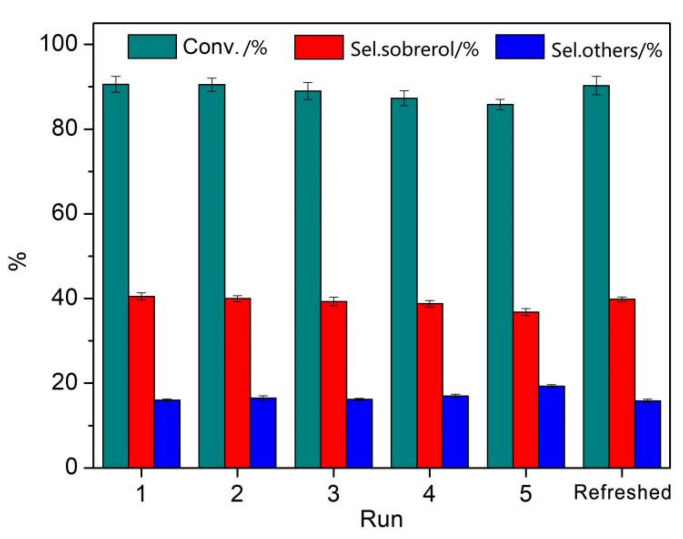
Reuse of AC-COIMI-NH_4_PW.

One of the advantages of using solid catalysts is that it is easy to separate and provide the possibility of direct reuse after recovery. In order to reduce the loss caused by the catalyst stuck on the filter paper and the difficulty to be entirely removed during filtration, the centrifugal method was used to recover the catalyst during the investigation of the catalytic stability of the specially prepared supported HPW catalyst AC-COIMI-NH_4_PW for reuse in the reaction of α-pinene with hydrogen peroxide solution as the oxidant. The mixture after the reaction was transferred to a centrifuge tube, centrifuged for 10 min on a high-speed centrifuge, and then poured out slowly the centrifuge tube after removing it. The solvent of the next round of reaction was used to wash down the catalyst on the wall of the centrifugal tube to the reaction bottle with the aid of ultrasound, followed by feeding it again, and carrying out a new round of reaction with the evaluation of the activity and selectivity of the recovered catalyst. The results of the catalytic stability investigation are described in Figure 8.

Reaction condition: α-pinene 10 mmol, acetonitrile 8 mL, H_2_O_2_ 20 mmol, catalyst 0.1 g, 40 °C, 3 h

In order to simply express the catalytic performance, only the conversion of α-pinene, Conv., reflecting the catalyst activity, the selectivity of sobrerol, S _sobrerol_, embodying the target of tandem epoxidation–hydration, and S _others_, the total selectivity of side reactions of α-pinene directly catalyzed by acid sites, are listed in the figure. It can be found that with the recycling of the catalyst, the conversion of α-pinene in the next round is reduced, the selectivity of epoxidation–hydration to sobrerol is also reduced in turn, while the total selectivity of acid-catalyzed isomerization is increased. The catalyst collected after the fifth reaction was washed with petroleum ether three times, dried in a vacuum at 60 °C and then weighed. It was found that the mass of the catalyst decreased from 100 mg to 97 mg, which was close to the reduction ratio of α-pinene conversion. It can be considered that the loss of catalyst was the main reason for the decline of the catalytic activity. The gradual increase of a strong acid site, for the isomerization of α-pinene, in recycling means that a small amount of the ammonium weak acid site NH_4_^+^ is transformed into a strong acid site H^+,^ which is derived from the hydrolysis of ammonium ion in the presence of water from the hydrogen peroxide aqueous solution. Based on this inference, the collected catalyst was treated by placing it in a desiccator filled with ammonia at room temperature for 24 h to restore H^+^ to NH_4_^+^ site on used AC-COIMI-NH_4_PW. After the regenerated catalyst was replenished to 100 mg with a fresh catalyst, the results of the sixth-reaction operation show that the catalytic activity and selectivity are restored as in the first run which is shown in the corresponding data of refreshed in Figure 8.

### 3.6. Catalytic Mechanism

Based on the epoxidation mechanism of perilla alcohol on supported vanadized phosphotungstic-acid catalysts [26], it is speculated that on the nitrogen-doped activated-carbon-supported ammonium phosphotungstic-acid catalyst AC-COIMI-NH_4_ PW, the catalytic reaction mechanism for the conversion of α-pinene and hydrogen peroxide into sobrerol through a series of “epoxidation–isomerization–hydration” reactions is as follows: hydrogen peroxide forms a three-center W=O_2_ peroxide with a tungsten oxide double-bond species W=O on the surface of the phosphotungstate ion.

**Figure 9:**
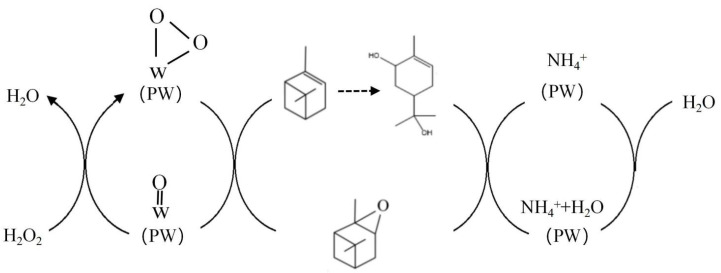
Catalytic cycle of α-pinene to sobrerol.

After being transported to the catalytic center in the solvent, the double bond of α-pinene is attacked with one of the oxygen atoms in W=O_2_, and C=C is oxidized to form an epoxy bond. The epoxy bond is adsorbed on the surface of AC-COIMI-NH_4_PW, and the dissociated H^+^ in the weak acid center NH_4_^+^ on AC-COIMI-NH_4_PW catalyzes the ring to open and form a tertiary carbocation at the C2 position. According to the β-decomposition rule, the tertiary carbocation at the C2 position causes the C-C bond between C1 and C7 in the quaternary ring to split, isomerizing to isopropyl tertiary carbocation outside the ring, which is then attacked by Lewis basic water molecules to undergo a hydration reaction. Finally, the product sobrerol is desorbed from the NH_4_^+^ center. The catalytic cycle of this process is shown concisely in Figure 9.

### 3.7. Comparison of Catalytic Performance with the Literature

Table 3 lists some literature reports on the catalytic synthesis of sobrerol in recent years. There are few works on the one-step synthesis of sobrerol from α-pinene through tandem reactions. Most people are concerned about the exploration of solid acid catalytic materials in the isomerization of α-epoxypinane to produce sobrerol. In terms of the selectivity of sobrerol, Costa and co-workers [20] prepared mesoporous Sn/SiO_2_ and Ce/SiO_2_ as catalysts with tetraethyl silicate (TEOS) as raw materials by the sol–gel method, which have the highest selectivity of sobrerol. Under the optimal conditions of acetone as the solvent, the conversion of α-epoxypinane is as high as 98%, and the selectivity of sobrerol is 72%. The catalytic isomerization of α-epoxypinane is carried out in solvents under mild reaction conditions, the conversion of α-epoxypinane can be achieved at over 90% within 2–5 h, and the conversion is almost complete in some case [22,23]. When using organic metal framework MIL-100 [30], acidic clay [31], Fe^2+^-MCM [32] or 12% Al SiO_2_ [33] as catalysts, although the reaction time is very short (even half an hour) to achieve a high conversion, the selectivity of sobrerol does not exceed 10%, which only exists as a byproduct. In this research work on the catalytic isomerization–hydration of α-epoxypinane to produce sobrerol, no experimental results were found to provide catalyst recovery and reuse, and the stability of these catalysts is unknown.

Compared with the catalytic systems studied in the literature using α-pinene and hydrogen peroxide as raw materials, the reaction temperature is 25 °C by V_2_O_5_ as catalyst [25], and the catalytic active species is peroxyvanadate, which is dissolved in the reaction system. The treatments of precipitation and washing are required at the end of the reaction, so the catalyst has no reusability, and its selectivity also is slightly lower than the nitrogen-doped activated-carbon-supported ammonium-phosphotungstate catalyst AC-COIMI-NH_4_PW in our this work. However, the active reaction temperature in vanadium containing MCM-41 [34] heterogeneous catalysis system is higher than that of AC-COIMI-NH_4_PW, and the selectivity of sobrerol is only 36.3%, significantly lower than that of AC-COIMI-NH_4_PW, with only four cycles of reuse. From this, it can be seen that AC-COIMI-NH_4_PW, the one-step synthesis of sobrerol from α-pinene and hydrogen peroxide, has high activity, good selectivity and excellent catalytic performance.

## 4. Conclusions

The surface of wooden activated carbon was oxidized and modified with the green oxidant hydrogen peroxide to increase the density of the carboxyl groups. Imidazole was bonded to form basic activated carbon doped with nitrogen on the surface. Phosphotungstic acid was fixed on the activated carbon through acid–base chemical adsorption, and then an ammoniated, supported with ammonium phosphotungstic acid, epoxidation-acid bifunctional catalyst AC-COIMI-NH_4_PW was constructed, by which a new process was developed for catalytic synthesis of sobrerol from α-pinene. This catalytic process exhibits outstanding advantages of green mild-reaction conditions, simple operation and stable catalytic performance and can be reused after recovery. A bifunctional catalytic cycle, W=O for epoxidation and NH_4_^+^ for hydration, on AC-COIMI-NH_4_PW is proposed for α-pinene with H_2_O_2_ conversion to sobrerol.

## Figures and Tables

**Table 1:** Effect of different catalysts on the oxidation of α-pinene with H_2_O_2_.

Cat.	Conv. /%	Sel. ^a^/%									
		1	2	3	4	5	6	7	8	9	Others ^b^
None	5.4	-	<0.1	4.2	37.1	37.3	5.8	2.2	1.6	0.4	11.4
AC	8.4	-	<0.1	4.2	32.6	38.3	6.8	2.8	1.8	0.4	13.0
AC-O	17.9	-	0.2	4.2	8.1	7.3	2.8	3.2	2.6	0.8	71.8
AC-COIMI	2.4	-	2.1	0.2	47.1	46.9	1.8	0.5	-	-	1.4
AC-COIMI-HPW	89.3	4.3	0.1	3.2	10.1	7.7	2.2	10.5.	4.3	2.1	55.8
AC-COIMI-NH_4_PW	90.6	2.6	0.8	7.5	8.6	8.3	5.6	40.5	6.6	3.4	16.0

Reaction condition: α-pinene 10 mmol, acetonitrile 8 mL, H_2_O_2_ 20 mmol, catalyst 0.1 g, temp. 40 °C, time 3 h. ^a^: **1**, 4-Acetyl-1-methyl-1-cyclohexene, **2**, α-Pinene epoxide, **3**, Campholenic aldehyde, **4**, Verbenol, **5**, Verbenone, **6**, Carveol, **7**, Sobrerol, **8**, 1,2-Pinanediol, **9**, 5-(2-hydroxypropan-2-yl)-2-methylcyclohex-2-en-1,4-diol. ^b^: others are the sums of monoterpene isomers.

**Table 2:** Effect of different solvents on the oxidation of α-pinene with H_2_O_2_.

Solvents ^a^	Conv. /%	Sel./%									
		1	2	3	4	5	6	7	8	9	Others
None	5.4	-	<0.1	4.2	37.1	37.3	5.8	2.2	1.6	0.4	11.4
DCM (1.60)	8.7	-	<0.1	4.7	40.6	21.2	<0.1	2.6	7.7	0.4	22.8
HAC (1.74)	31.5	-	<0.1	11.8	3.2	17.1	1.7	19.1	8.9	2.3	35.9
EA (1.78)	22.1	-	<0.1	10.7	24.6	22.1	4.3	18.8	4.6	1.3	13.6
THF (1.63)	52.0	6.9	11.6	7.5	14.1	31.9	1.2	9.7	3.1	0.9	13.1
Dioxane (0)	45.3	4.6	12.3	8.2	15.8	27.4	2.3	8.3	2.5	2.2	16.4
Acetone (2.88)	66.2	3.3	<0.1	16.0	4.8	8.8	3.1	28.8	6.7	3.8	24.8
Butanone (2.78)	46.1	9.6	10.0	6.7	11.8	2.1	2.0	24.5	11.	5.1	12.9
CAN (3.92)	90.6	2.6	0.8	7.5	8.6	8.3	5.6	40.5	6.6	3.4	16.0

Reaction condition: α-pinene 10 mmol, solvent 8 mL, H_2_O_2_ 20 mmol, catalyst 0.1 g, 40 °C, 3 h. ^a^: Dipole moment (D) in parentheses.

**Table 3:** Comparison of reported catalytic synthesis results of sobrerol from α-pinene or α-epoxypinane.

Substrate/Catalyst	T/°C	t/h	Conv.%	Sel./%	Times ^a^	Ref.
α-Pinene/AC-COIMI-NH_4_PW	40	3	90.6	40.5	6	This work
α-Epoxypinane/Ce/SiO_2_	25	3	98	72	1	[20]
α-Epoxypinane/HPW	60	3	100	38	1	[22]
α-Epoxypinane/CsPW	25	5	100	48	1	[23]
α-Pinene/V_2_O_5_	25	2	85.7	38.0	1	[25]
α-Epoxypinane/MIL-100	30	0.5	98	7	1	[30]
α-Epoxypinane/acidic clays	50	2	99.9	8.3	1	[31]
α-Epoxypinane/Fe^2+^-MCM	40	3	96	56	1	[32]
α-Epoxypinane/12%Al-SiO_2_	30	0.5	80	8	1	[33]
α-Pinene/V-M(x)	70	7	84.5	36.3	4	[34]

^a^: The numeral referring to total times in recycling use of typical catalyst.

## Data Availability

The data presented in this study are available on request from the corresponding author.
